# Comparing the consequences of natural selection, adaptive phenotypic plasticity, and matching habitat choice for phenotype–environment matching, population genetic structure, and reproductive isolation in meta‐populations

**DOI:** 10.1002/ece3.3816

**Published:** 2018-03-13

**Authors:** Marion Nicolaus, Pim Edelaar

**Affiliations:** ^1^ Department of Molecular Biology and Biochemistry Engineering University Pablo de Olavide Sevilla Spain; ^2^ Conservation Ecology Group, Groningen Institute for Evolutionary Life Sciences (GELIFES) University of Groningen Groningen The Netherlands

**Keywords:** biased dispersal, genetic structure, individual‐based simulation model, local adaptation, meta‐population, speciation

## Abstract

Organisms commonly experience significant spatiotemporal variation in their environments. In response to such heterogeneity, different mechanisms may act that enhance ecological performance locally. However, depending on the nature of the mechanism involved, the consequences for populations may differ greatly. Building on a previous model that investigated the conditions under which different adaptive mechanisms (co)evolve, this study compares the ecological and evolutionary population consequences of three very different responses to environmental heterogeneity: matching habitat choice (directed gene flow), adaptive plasticity (associated with random gene flow), and divergent natural selection. Using individual‐based simulations, we show that matching habitat choice can have a greater adaptive potential than plasticity or natural selection: it allows for local adaptation while protecting genetic polymorphism despite global mating or strong environmental changes. Our simulations further reveal that increasing environmental fluctuations and unpredictability generally favor the emergence of specialist genotypes but that matching habitat choice is better at preventing local maladaptation by individuals. This confirms that matching habitat choice can speed up the genetic divergence among populations, cause indirect assortative mating via spatial clustering, and hence even facilitate sympatric speciation. This study highlights the potential importance of directed dispersal in local adaptation and speciation, stresses the difficulty of deriving its operation from nonexperimental observational data alone, and helps define a set of ecological conditions which should favor its emergence and subsequent detection in nature.

## 1 INTRODUCTION

Organisms commonly experience significant spatiotemporal variation in their physical or social environment. In response, different mechanisms may act that enable organisms to cope with such environmental heterogeneity. In this study, we focus on three very distinct routes toward increased local performance: phenotypic plasticity (Schlichting & Pigliucci, 1998), divergent natural selection (Schluter, 2001), and matching habitat choice (Edelaar, Siepielski, & Clobert, 2008). These mechanisms can each lead to improved performance but their ecological and evolutionary impacts can differ greatly. This study primarily aimed to illustrate in a heuristic, proof‐of‐concept manner (Servedio et al., 2015) the similarities and differences among these mechanisms in terms of consequences for the population. We do not discuss how these mechanisms evolve and interact, as this is addressed elsewhere (e.g., Edelaar, Jovani, & Gomez‐Mestre, 2017). Instead we provide a direct comparison of their impacts on meta‐population structure and functioning, something that has not been undertaken before (Jacob, Bestion, Legrand, Clobert, & Cote, 2015). Secondarily, we hope to attract further research attention to the overlooked phenomenon of matching habitat choice in view of its potential evolutionary and ecological impacts.

The degree to which organisms obtain a better match between their pheno‐/genotype and the environment may vary depending on the underlying mechanism used to this end, and on the interaction among evolutionary forces (Blanquart, Kaltz, Nuismer, & Gandon, 2013; Kawecki & Ebert, 2004). Phenotypic plasticity, that is, the ability of a genotype to adaptively alter its phenotype in response to environmental variation (Dewitt & Scheiner, 1994), has been proven to be a powerful and quick solution to the problem of individual adaptation to heterogeneous environments (Ghalambor, Mckay, Carroll, & Reznick, 2007) providing it is not too costly (Auld, Agrawal, & Relyea, 2010; Dewitt, 1998). This mechanism may be particularly important for the persistence of nonmobile organisms that cannot escape local or changing conditions. For example, in many plant species, changes in light or humidity conditions trigger adaptive changes in leaf or root morphology (Sultan, 2000), but examples abound across all kingdoms of life.

When plasticity is not possible or insufficient, adaptation of populations may be achieved via genetic changes resulting from spatially divergent natural selection (Kawecki & Ebert, 2004), sometimes even at fine spatial scales (Richardson, Urban, Bolnick, & Skelly, 2014). Yet, the degree to which adaptive population genetic divergence is achieved is largely dependent on the interplay between natural selection and dispersal that can swamp the effect of selection by introducing maladapted foreign alleles into locally adapted populations (Barton & Partridge, 2000; Kawecki & Ebert, 2004; North, Pennanen, Ovaskainen, & Laine, 2011). In classic evolutionary theory, dispersal is assumed to be random with respect to genotypes, implying that local adaption will only occur when selection exceeds the homogenizing effect of gene flow (Edelaar & Bolnick, 2012; Kawecki & Ebert, 2004; Lenormand, 2002; Richardson et al., 2014). Constraining effects of gene flow on adaptive divergence have been documented across diverse taxa (e.g., fish: Hendry, Taylor, & McPhail, 2002; plants: Paul, Sheth, & Angert, 2011; birds: Postma & van Noordwijk, 2005; amphibians: Storfer, Cross, Rush, & Caruso, 1999) suggesting that dispersal may commonly limit adaptation by natural selection in nature (Räsänen & Hendry, 2008).

However, the hampering role of gene flow in the process of local adaptation by natural selection has recently been challenged by the growing awareness that dispersal is often nonrandom with respect to genotype (e.g., Clobert, Le Galliard, Cote, Meylan, & Massot, 2009; Cote, Clobert, Brodin, Fogarty, & Sih, 2010; Edelaar & Bolnick, 2012; Edelaar et al., 2008; Stevens et al., 2014). Such nonrandom dispersal can involve genotypes evaluating environmental variation and settling in the habitat where they will perform best given their phenotype (Bolnick & Otto, 2013; Edelaar et al., 2008; Ravigné, Dieckmann, & Olivieri, 2009). Such “matching habitat choice” thus allows individuals to be an *agent* instead of a *target* of selection and thereby exerts a distinct evolutionary force that can lead to adaptive evolution, even in the absence of natural selection (Bolnick & Otto, 2013; Edelaar & Bolnick, 2012). Empirical evidence for matching habitat choice is still limited, yet, some studies have shown that, for example, different phenotypes disperse and settle preferentially in habitats where they are more camouflaged (Dreiss et al., 2012; Gillis, 1982; Karpestam, Wennersten, & Forsman, 2012; Rodgers, Gladman, Corless, & Morrell, 2013) or more physiologically adapted (Bestion, Clobert, & Cote, 2015; Jacob et al., 2017).

The interplay between spatially divergent selection, plasticity, and random versus directed dispersal not only affects local performance, but can also affect the genetic diversity and structure of populations (Arendt, 2015). Unconstrained phenotypic plasticity (i.e., without costs) should lead to adaptive phenotypic differentiation among populations exposed to different environments without affecting the genetic composition of populations. In contrast, divergent natural selection and matching habitat choice should reduce standing genetic variation within locally adapted populations (through selective removal of genotypes or directed dispersal), but increase genetic differentiation and genetic variance at the meta‐population scale (Hedrick, 2006; Hedrick, Ginevan, & Ewing, 1976). Nonetheless, the effect of gene flow on population genetic diversity and structure in these cases can vary radically: strong gene flow should *homogenize* the genetic pool of the meta‐population and erode its genetic structure when it is random (Endler, 1973; Hendry, Day, & Taylor, 2001; Lenormand, 2002; Slatkin, 1987), while it should *enhance* genetic structure and protect polymorphism in the meta‐population when it is nonrandom (Berner & Thibert‐Plante, 2015; Edelaar & Bolnick, 2012) as in matching habitat choice (Armsworth, 2009; Armsworth & Roughgarden, 2005; Edelaar et al., 2008).

**Figure 1:**
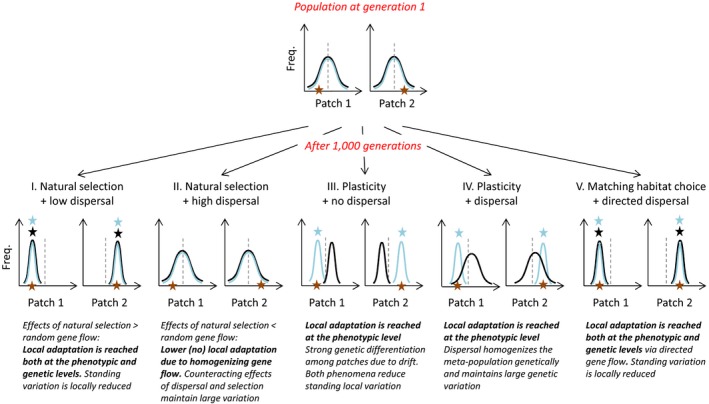
Conceptual representation of changes in the genotypic (black) and phenotypic (blue) frequency distribution over time (in the absence of mutation) for two patches that differ in their “*environment*” value (orange star). This is performed under five distinct scenarios: (I) natural selection with little and random gene flow, (II) natural selection with much and random gene flow, (III) adaptive phenotypic plasticity in the absence of random gene flow, (IV) adaptive phenotypic plasticity in the presence of random gene flow, and (V) matching habitat choice where gene flow is directed. The blue and black stars indicate where adaptive phenotypic or genotypic match is achieved, respectively. A reduction in the width of the distribution indicates a decrease in standing genetic variation, while a shift in the mean indicates a change in the population genetic structure. Note that scenarios I and V yield the same pattern, despite a very different underlying mechanism. Also note that dispersal disrupts adaptation, but only if it is random with respect to genotype

The evolutionary and ecological implications of plasticity, divergent selection, and matching habitat choice have been studied empirically and theoretically (e.g., Agrawal, 2001; Bolnick & Otto, 2013; Edelaar et al., 2008; Jacob et al., 2015; Pfenning et al., 2010; Price, Qvarnström, & Irwin, 2003; Ravigné et al., 2009) but rarely in combination in the same study (but see Edelaar et al., 2017; Scheiner, 2016). Such formal comparison is needed because similar patterns of adaptation or population structure may arise from different combinations of environments and mechanisms, and not distinguishing between them can lead to misinterpretation and misunderstanding of the biological processes that underlie certain empirical patterns. To highlight these similarities as well as differences in the effects of the three compared mechanisms, we use a simplified individual‐based simulation modeling approach in a meta‐population framework. We explore how the degree of local adaptation (at the phenotypic and genotypic level) and the population genetic structure (for functional and neutral loci) depend on the interactions between (1) the different mechanisms to increase local performance (contrasting the lesser‐known effects of matching habitat choice to the well‐known effects of divergent natural selection and adaptive plasticity), (2) the degree (low vs. high) and kind (random vs. nonrandom) of dispersal, (3) the temporal variability of the environment (mild vs. strong), and (4) the spatial scale of mating (local vs. global). We aim for our models to be simple and heuristic rather than realistic; therefore, we model and compare the consequences of these adaptive solutions under five extreme scenarios (Figure 1).

## 2 MATERIALS AND METHODS

The model we used to explore the above topics is an adaptation of the freely available model developed by Edelaar et al. (2017) to study the evolution and interaction between population genetic divergence, phenotypic plasticity, and matching habitat choice. Edelaar et al. (2017) found that plasticity and matching habitat choice are both very effective in adapting organisms to temporal heterogeneity, and each by itself readily evolves even in the presence of moderate costs. However, when combined, matching habitat choice generally only evolves when plasticity becomes too costly or is otherwise constrained. Building upon Edelaar et al. (2017), we here simply define fixed sets of conditions and trait states that best characterize and favor each of the three compared mechanisms.

### 2.1 Model components: Environments and individual characteristics

#### 2.1.1 The environment

As in Edelaar et al. (2017), our model aims at capturing and understanding general patterns that are not necessarily biologically realistic yet empirically relevant and interpretable, based on features that we considered to be important a priori. The model starts with a population of 1,000 individuals randomly distributed among 100 habitat patches characterized by an environmental value “*environment*” (see Table S1 for description of model components) and each patch receiving 10 individuals. The “*environment*” trait is modeled as a vector with a range of 0–360 degrees (Table S1). This is a common approach in physics (and similar to the modeling of a torus‐shaped spatial world) that helps to model various degrees of environmental predictability (temporal autocorrelation), and to avoid boundary effects and changes in the variability across patches over time. As the environment value changes stochastically and does not have a central tendency, it does not allow adaptation by a generalist genotype, which helps us to increase the contrast between divergent natural selection and the other two mechanisms. At model initiation each patch receives a random draw from the range 0–360 but after that patch “*environment*” value changes in time. There is no spatial structure to the patches (see visual examples in Figure S2). This is similar to the classical infinite island model as used in population genetics where migration rates are identical between all populations (Wright, 1943).

#### 2.1.2 Individual traits

Individuals are sexual hermaphrodites and inherit six traits from their two parents: (1) a “*genotype*” for a functional quantitative trait, used to assess adaptation; (2) a “*neutral genotype*” for a neutral quantitative trait, used to measure neutral divergence (for details on inheritance see below). To allow easy tracking of matching with patch “*environment*” values, “*genotype*” and “*neutral genotype*” also have a circular distribution (range 0–360 degrees; Table S1, Figure S2). Further inherited traits are: (3) a “*plasticity potential*” trait that determines an individual's ability to produce a range of phenotypes; (4) a “*plasticity habitat sensitivity*” trait that determines an individual's ability to gather and process information about its local environment as used to develop the best matching phenotype; (5) a “*dispersal potential*” trait that determines the number of patches an individual will prospect prior to dispersal; and (6) a “*dispersal habitat sensitivity*” trait that determines an individual's ability to gather and process information about the environment in all prospected patches as used to choose the best‐matching habitat patch (Table S1). These last four traits have a range from 0 to 1. Note that hereby both plasticity and matching habitat choice are made up of two separate traits (traits 3 and 4 vs. 5 and 6, respectively). This allows us to easily change between random and nonrandom dispersal (by varying “*dispersal habitat sensitivity*”) independent of dispersal rate (“*dispersal potential*”). Also note that the conceptual similarity in design among those four traits makes the comparison between plasticity and matching habitat choice straightforward and unbiased. At start, individuals receive a random value for their “*genotype*” and “*neutral genotype*” and specific values for the other four inherited traits, which depend on the specific scenario we wanted to model (see Table S1). All individual trait values and the “*environment*” are thus initially uncorrelated. Unlike the model by Edelaar et al. (2017), there are no costs associated with any of the traits described here as we are not interested in how they affect the results, and they showed that all mechanisms can evolve in the presence of small to moderate costs anyway.

### 2.2 Basic loop: Elements of each generation

**Figure 2:**
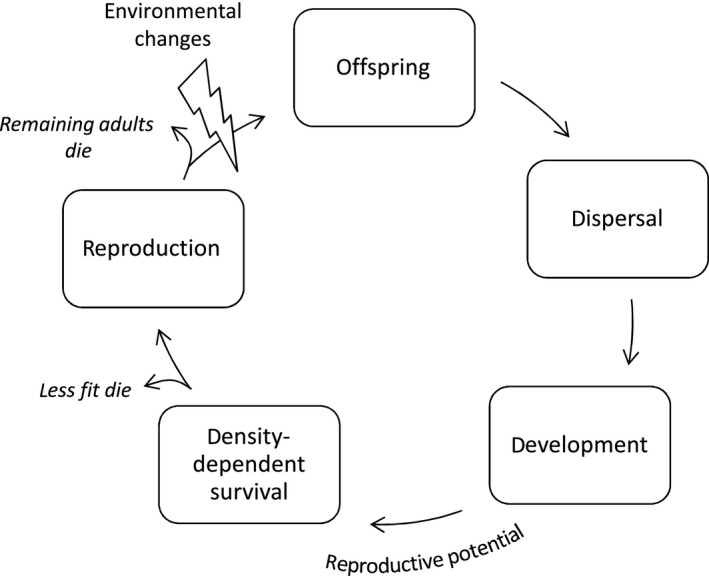
Basic loop of the model. First juveniles are born. Offspring then disperse and develop. After this, their reproductive potential is calculated according to their phenotypic mismatch with the local habitat. If the local density is higher than carrying capacity, the excess individuals with the lowest reproductive potential die. Subsequently individuals reproduce (with mutation) relative to their reproductive potential. Sexual reproduction is either local (within the local patch) or global (across the meta‐population). Remaining adults die. Finally, the environment changes (either mild and predictable or strong and unpredictable) and the process starts again with the birth of new offspring

The simulation is in discrete time. Each model is run for 1,000 generations that do not overlap and each simulation (of 1,000 generations) is run independently with 10 different initial seed numbers (10 independent replicates under the same parameter settings, to assess stability of results). Each generation, the following operations are performed (see also Figure 2):

#### 2.2.1 Birth of offspring

The continuous traits of the offspring are used as quantitative traits, but not modeled by a number of separate loci. Therefore, the value for an offspring is simply calculated as the average of the two parental traits. This excludes segregation variance in the offspring, setting aside this source of genetic variance. While obviously this is not a realistic assumption, it helps to isolate and clarify the effects of the modeled mechanisms on genetic structure and variability. Also note that while this would result in regression to the mean (and loss of genetic variation) for a linear trait, this in principle is not the case for a circular trait as used here (which has no meaningful mean value). Inheritance occurs without any mutation, except for “*genotype*” and “*neutral genotype*” which are inherited with a mutational effect that is modeled by extracting a pseudorandom number from a negative exponential distribution (Eyre‐Walker & Keightley, 2007) characterized by a “*mean mutational change*” of 0.01, adding or subtracting it from each inherited trait with equal probability and multiplied by 180 (the maximum change possible for a circular trait) (Table S1). As the intermediate value for two parents with genotypes of, for example, 4 and 358 degrees is not simply the arithmetic mean (it should be 1 degree and not 181 degrees), we used circular statistics to calculate a new offspring's genotypic value (θ) (Jammalamadaka & SenGupta, 2001) as:

$$\theta =\text{arctan}\left(\frac{\sin\;\overset{¯}{a}}{\cos\;\overset{¯}{a}}\right)+\mu$$

where $\cos\;\overset{¯}{a}=\frac{X}{r},\;\sin\;\overset{¯}{a}=\frac{Y}{r},\;r=\sqrt{X^{2}+Y^{2}},\;X=\frac{\sum_{i=1}^{2}\cos\;a}{2}\;\text{and}\;Y=\frac{\sum_{i=1}^{2}\sin\;a}{2}.$

#### 2.2.2 Dispersal

Individuals first define a set of habitat patches to prospect depending on their “*dispersal potential*,” which simply equals the probability with which any patch is included in this set. Then they use their “*dispersal habitat sensitivity*” to assess the “*environment*” of each patch, including the natal patch. When this sensitivity has the minimum value of 0 they assign a random environmental value to each patch, whereas when it has the maximal value of 1, the assessment of the environment of each assessed patch is completely accurate (intermediate values for this trait will not be modeled here, so are of no further concern). Subsequently individuals disperse to the patch (including staying in the home patch) that they estimate as suiting them best, that is, to the patch with the lowest “*mismatch*” between their “*phenotype*” and the perceived “*environment*” value. Hence, an effective ability for matching habitat choice is characterized by high values for “*dispersal potential*” and “*dispersal habitat sensitivity*.”

#### 2.2.3 Development

At birth, the individual phenotypic value equals its genotypic value. However, after dispersal, individual first use their “*plasticity habitat sensitivity*” to assess the “*environment*” of their settlement patch in the same way as with “*dispersal habitat sensitivity*.”

Individuals then use their “*plasticity potential*” to alter their phenotype as close as possible to the perceived “*environment*” value of the patch. Hence, effective phenotypic plasticity is characterized by high values for “*plasticity habitat sensitivity*” and “*plasticity potential*.” In this study, individuals disperse first and then develop; this order of life‐history stages does not affect model outputs as plasticity and dispersal are mutually exclusive, except for scenario IV. As scenario IV models adaptive plasticity with random dispersal, dispersal is modeled first. (For the minor influence of the order of life‐history stages when adaptive plasticity and adaptive habitat choice are not exclusive, see Edelaar et al., 2017.)

#### 2.2.4 Survival

Adult survival is density dependent (to maintain population size constant) and selective (to promote local adaptation and minimize the importance of genetic drift): when population size in a patch exceeds 10 individuals, only the ten individuals with the highest “*reproductive potential*” survive. Reproductive potential is calculated as:

$${}^{\prime \prime }reproductive\;potential^{\prime \prime }=1{-}^{\prime \prime }mismatch^{\prime \prime }$$

with “*mismatch*” being the absolute difference between the true value of the “*environment*” and the “*phenotype*” of an individual, divided by 180 (maximum difference in degrees possible for a circular trait):

$${}^{\prime \prime }mismatch^{\prime \prime }={\left(\right|}^{\prime \prime }environment^{\prime \prime }{-}^{\prime \prime }phenotype^{\prime \prime }\left|\right)/180$$

“*Reproductive potential*” thus varies from 1 (perfectly locally matching phenotype) to 0 (worst possible, completely nonmatching phenotype). This fitness function is linear (instead of, e.g., Gaussian) because we do not wish to vary the strength of selection as a separate parameter. The “*mismatch*” will increase when “*plasticity potential*” or “*dispersal potential*” are too low to achieve that its “*phenotype*” is locally optimal, or if the individual makes an incorrect assessment of the “*environment*” (e.g., when their “*plasticity‐*” or “*dispersal habitat sensitivity*” value is low). And the latter case it can even express maladaptive developmental plasticity or habitat choice (see steps 2 and 3).

#### 2.2.5 Reproduction

Surviving individuals reproduce according to their reproductive potential. If individual “*reproductive potential*” is greater than a randomly drawn number between 0 and 1, reproduction is allowed. This stochastic reproduction is repeated three times, with the same partner. Preliminary simulations showed that this level of fecundity was necessary to maintain viable populations, particularly in scenarios where dispersal was not allowed. When reproduction is local, individuals reproduce with a random mate from within their patch. In this case, reproduction is only allowed if the number of individuals present in the patch is ≥2 (otherwise the local population goes extinct). When reproduction is global, individuals reproduce with a random mate chosen from the entire meta‐population. Note that *reproductive potential* thus is selected upon twice: during density‐dependent population regulation, and during reproduction. This further reduces the effect of genetic drift. Adults die immediately after reproduction, so generations are nonoverlapping.

#### 2.2.6 Environmental changes

After reproduction, the “*environment*” changes for each patch independently. In a first set of simulations, this happens according to a random draw from a normal distribution characterized by a mean of zero and a standard deviation of 10 degrees, that is, we modeled consistent (predictable) mild temporal change. In the second set of simulations, only two opposite types of “*environment*” exist and each generation these values are randomly re‐assigned to each patch, that is, we modeled strong, unpredictable temporal change.

### 2.3 Scenarios of adaptation

We modeled our three main mechanisms to cope with environmental change (i.e., population genetic divergence by natural selection, individual adaptive phenotypic plasticity, and individual matching habitat choice) by combining different values for the inherited dispersal and plasticity traits such that these would create the desired scenario (see Table S1). Again, because our goal was not to study the conditions under which these mechanisms evolve but to study their consequences for the meta‐populations, we chose those trait values that define each scenario (e.g., adaptive plasticity has “*plasticity potential*” and “*plasticity habitat sensitivity*” at 1, but “*dispersal habitat sensitivity*” at 0). Dispersal potential needed some additional choices. As matching habitat choice benefits from a high “*dispersal potential*,” we have fixed this trait to 1. For divergence by natural selection, however, we have allowed “*dispersal potential*” to evolve within a range of either low (0–0.1) or high (0.9–1) values in order to model the effects of weak versus strong random gene flow on local adaptation. For adaptive plasticity, populations either had no dispersal or were allowed to evolve any values of “*dispersal potential*” in order to relax constraints imposed by restricted movements and to focus solely on the output of adaptive plasticity. As explained above, on top of these 5 basic scenarios we also varied the mode of reproduction (local or global, see point 5.) and the level of environmental variation (continuous, predictable vs. binary, unpredictable, see point 6.), yielding a total of 20 distinct sets of simulations. Modeling different scales of reproduction in combination with different modes of dispersal is important as both influence gene flow: low dispersal, matching habitat choice and local reproduction should promote genetic divergence whereas high dispersal and global mating should promote genetic homogenization. Hence, it becomes particularly important to compare the population consequences of the interacting effects of random versus directed dispersal and the scale of reproduction to evaluate under which conditions adaptive genetic divergence is achieved and maintained. Because our interest is to investigate the consequences of distinct responses to environmental heterogeneity on population genetic structure, we only use these extreme combinations of trait values and did not allow the dispersal and plasticity traits to evolve (except for moderate evolution of dispersal potential as explained above).

### 2.4 Quantifying output population characteristics

From each model simulation run, we extracted and inferred several parameters characterizing the population after 1,000 generations (when equilibrium had been reached).

#### 2.4.1 Degree of local adaptation (mismatch)

To measure the degree of local performance at the phenotypic and genetic level, we calculated the mean population phenotypic (Δ_p_) and genotypic (Δ_g_) mismatch, that is, the mean absolute difference between individuals’ “*phenotype*” or “*genotype*” and the “*environment*” value of their settlement patch, as:

$$\Delta_{p}=\frac{1}{N}\sum_{j=1}^{N}\bar{\Delta_{\mathrm{pj}}}\;\text{and}\;\Delta_{g}=\frac{1}{N}\sum_{j=1}^{N}\bar{\Delta_{\mathrm{gj}}}$$

with $\bar{\Delta_{\mathrm{pj}}}$ being the mean phenotypic mismatch of individuals of a patch *j*, $\bar{\Delta_{\mathrm{gj}}}$ the mean genotypic mismatch of individuals of a patch *j*, and *N* the total number of occupied patches in the meta‐population. Δ_p_ Measures if the phenotypes on average are well matched with their local environments (irrespective of their genotypes), Δ_g_ does the same but then measured at the level of the genotype.

#### 2.4.2 Population genetic structure

First we estimated the functional (and neutral) genotypic variance within (σ²_*W*_) and between (σ²_*B*_) patches and calculated an index “*Q*st” (or “neutral *Q*st”) as:

$$Q\text{st}=\frac{\sigma_{B}^{2}}{\sigma_{B}^{2}+2\times \sigma_{W}^{2}}$$

“*Q*st” and “neutral *Q*st” (n*Q*st) are thus comparable to the index *Q*st as used in quantitative genetics to quantify the degree of population genetic structure, that is, of differentiation at quantitative traits (Spitze, 1993) with the difference that here the underlying trait distribution is not necessarily normal. A *Q*st value close to 1 indicates very strong population differentiation (relatively low within‐population variance), a value close to 0 indicates very weak differentiation (relatively low between‐population variance), whereas having equal variances within and among patches ($\sigma_{W}^{2}=\sigma_{B}^{2}$) yields a *Q*st value of 0.33.

### 2.5 Model implementation

The source code of the model used here is available in the Supporting Information (SI 4—Data S1). The model is implemented in Netlogo 5.0.5 (Wilensky, 1999). NetLogo is freely downloadable from http://ccl.northwestern.edu/netlogo/download.shtml. Our open source model when opened in NetLogo has a graphical interface that allows users to easily change the settings of several parameters (even during a simulation run) and shows in real‐time summary graphs of several output variables. Downloading our model therefore allows corroboration of our conclusions, further investigation, or use in outreach or teaching. All the population output parameters have been calculated in R v.3.2.0 (R Development Core Team, 2015) using the *RNetlogo* and *circular* packages. Because we do not know if the underlying distribution for the population output parameters is a normal distribution, we used the “sim” function of the *arm* package to simulate values of the posterior distribution of the output parameters. 95% Credible intervals (CrI) around the mode were extracted based on 10 simulations of 1,000 generations of each model as the 2.5% and 97.5% quantiles of the posterior distribution of parameter estimates.

## 3 RESULTS

### 3.1 Degree of local matching

**Figure 3:**
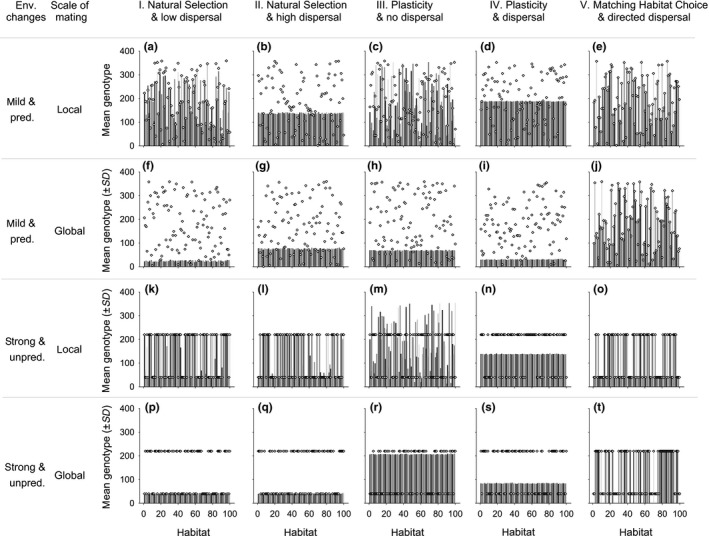
Examples of the output of one run after 1,000 generations. Mean genotype in each of 100 habitat patches (*x*‐axis) is represented by grey bars, while the habitat “*environment*” value is indicated by white diamonds. Results are depicted for each of the five modeled scenarios (columns I–V) with either local mating (i.e., within their habitat patch) or global mating (i.e., at the scale of the meta‐population) and with either mild predictable or strong unpredictable (random shifts between 40° and 220°) environmental changes

**Figure 4:**
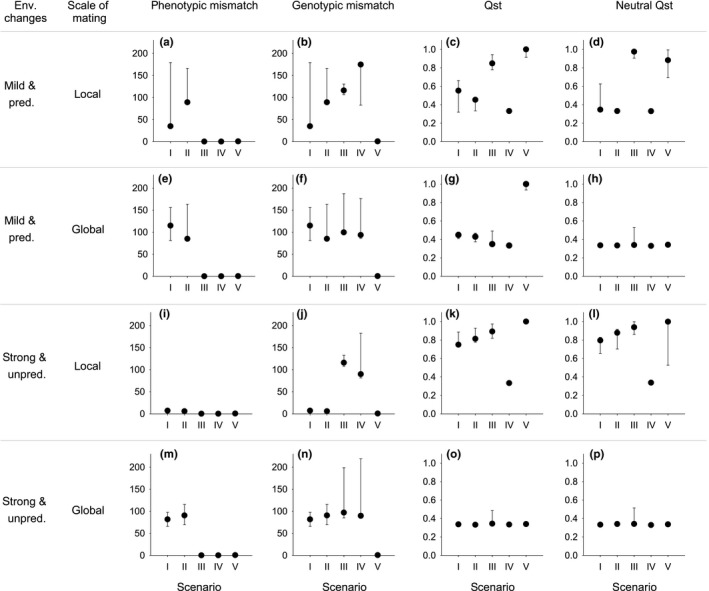
Consequences of the modeled scenarios for the meta‐population in terms of mismatch of individuals (absolute difference between the habitat “*environment*” value and their “*phenotype*” and “*genotype*”) and population genetic structure for functional (*Q*st) and neutral (neutral *Q*st) traits. Posterior distributions of the simulation results (10 independent runs) as represented by their modes and 95% credible intervals are depicted for each of the five scenarios of local adaptation (I–V), with either local or global mating and with either mild predictable or strong unpredictable environmental changes

Plasticity and matching habitat choice reached the same degree of phenotype–environment match, with basically no remaining mismatch. This was independent of the degree of temporal environmental change (Table S4, Figure 4a,e,i,m). In contrast, for natural selection both random dispersal (i.e., movement of individuals) and global mating (i.e., movement of gametes) disturbed phenotypic matching when environmental change was mild (Table S4, Figures 3a,b,f,g and 4a,e). When environments changed strongly and the scope for selection increased, natural selection improved the phenotypic matching of populations when mating was local and it reached levels similar to plasticity and matching habitat choice, even when dispersal was high (Table S3, Figure 4i). In contrast, when mating was global, local adaptation via natural selection was not possible (Table S3, Figure 4m). At the genotypic level, the results were exactly the same, except that for plasticity there was never any genetic adaptation (Table S3, Figure 4b,f,j,n). Matching habitat choice consistently reached high levels of phenotypic and genotypic adaptation, independent of the degree of environmental change or the scale of mating (Table S3).

When the “*environment*” value alternated randomly between two extreme values and individuals reproduced globally, most mismatch values converged to 90 (i.e., 180 degrees/2) (Table S3, Figure 4m,n), except for matching habitat choice where mismatch always remained closed to 0 (Table S3, Figure 4m,n). This convergence toward 90 is due to the fact that genetic variation was lost and only a single genotype became fixed across the entire meta‐population (Figure 3p–s), and consequently had a 50% chance of being locally matching. In contrast, with matching habitat choice also one of the two potentially adaptive genotypes became fixed in the population, but it actively avoided settling in the “wrong” patch (Figure 3t), thereby maintaining local adaptation (Figure 4m,n) but leaving half of the patches empty (Figure 3t).

### 3.2 Population genetic structure

Under mild environmental changes, our results confirm that random dispersal (scenarios I, II, IV) and reproduction at a global scale are two homogenizing phenomena that erode genetic structure both for a functional and a neutral trait (low *Q*st and n*Q*st values, Table  S3, Figures 3 and 4c–d,g–h). However, nonrandom dispersal as in matching habitat choice, maintained a *Q*st value close to 1, at any scale of reproduction (Figure 4c,g). In fact, when global mating basically eroded all genetic variation in the meta‐population across all other scenarios (Figure 3f–i), matching habitat choice maintained adaptive genetic variation at the meta‐population level (Figures 3j and 4g). In contrast, global reproduction did reduce n*Q*st for all scenarios (Figure 4h).

When the “*environment*” value alternated randomly between two extreme values, the genetic structuring of the meta‐population increased in the natural selection scenarios I and II with local reproduction, even in the presence of high dispersal among patches (Table S3, Figure 4k vs. 4c). The natural selection process, however, maintained the production of “hybrids” (intermediate offspring of two opposite parental genotypes) which explains why the *Q*st value under scenarios I and II remained lower than the *Q*st value under matching habitat choice which prevented the formation of intermediate, hybrid genotypes (Table S3, Figures 3k,l,o and 4k). In contrast, when reproduction was global, genetic mixing swamped adaptation to local conditions, single genotypes became (nearly) fixed, and *Q*st values of all scenarios converged to low values (Table S3, Figure 4o). For the scenarios involving natural selection or matching habitat, which of the two adaptive genotypes fixed was probabilistic; for the scenarios with plasticity a random genotype became fixed.

## 4 DISCUSSION

This study aimed at comparing directly the ecological and evolutionary population consequences of three distinct mechanisms to cope with environmental heterogeneity. While our results reassuringly confirm some well‐known aspects (e.g., random gene flow disrupts local adaptation), it also highlights some very important consequences that are specific to matching habitat choice (our main interest here) as compared to plasticity and selection: (1) it is the only mechanism that consistently shows adaptation at the phenotypic and genotypic level, (2) it generates higher *Q*st values (=higher population genetic structure; except under global mating and strong unpredictable environmental changes), (3) it has a greater capacity to maintain genetic variation at the meta‐population level, (4) it is more effective in preventing the mating between locally adapted and maladapted individuals. We discuss below in greater detail the evolutionary and ecological implications of our results.

### 4.1 Adaptation, population genetic structure, and evolutionary stability of meta‐populations

Many empirical and theoretical studies have investigated the constraining role of random gene flow in the local adaptation process. It is generally predicted that adaptive population divergence can only be achieved when the effects of selection are stronger than the homogenizing effects of random dispersal (Kawecki & Ebert, 2004; Lenormand, 2002). Unaffected by our simplifying (and therefore to some extent unrealistic) model assumptions, our simulations confirmed that this is the case: when the strength of selection was moderate (i.e., under mild and predictable environmental changes), a high rate of genetic mixing (occurring either via random dispersal and/or random mating at a global scale) depleted the genetic variance of the meta‐population, thereby preventing local adaptation (genotypic mismatch varied from moderate to maximal values). In contrast, when the strength of selection increased (i.e., with strong and unpredictable environmental changes), effects of selection as expected outweighed the homogenizing effects of random gene flow, resulting in the maintenance of adaptive genetic variance at the meta‐population scale.

In contrast, both adaptive plasticity and matching habitat choice showed a higher capacity to achieve adaptation to local environments. This matches expectations, as the values used for “potential” and “sensitivity” traits were highly favorable, but importantly, also because these mechanisms allow locally mismatched individuals to improve their performance (i.e., it enables adaptation at the individual level), whereas selection only operates toward their elimination (i.e., it enables adaptation only at the population level). It is for this reason that individually flexible responses to environmental heterogeneity, such as plasticity and habitat choice, evolve via natural selection: they allow for an increase in fitness in otherwise locally maladapted individuals (Edelaar et al., 2017). Basically, their evolution reduces the scope for natural selection, and in the extreme case may result in the virtual elimination of it (i.e., no more selective mortality and reproduction).

Given the higher capacity of matching habitat choice and adaptive plasticity to achieve adaptation to local environments, this implies that their evolution promotes evolutionary stability and persistence of meta‐populations. Likewise, our findings support that matching habitat choice and plasticity are more likely to occur in meta‐populations exposed to fast and/or strong environmental changes, because these mechanisms allow for quicker adaptive responses to environmental changes at the within‐generation individual level, instead of at the between‐generation population level as for natural selection. Given the unprecedented rate of environmental changes of our current time (e.g., habitat fragmentation, Fahrig, 2003; or climate change, Walther et al., 2002), disturbed populations may thus increasingly rely on matching habitat choice and plasticity to adapt and survive these changes. This is broadly recognized for plasticity, but attention for matching habitat choice has been very limited. The recent findings that in common lizards (*Zootoca vivipara*) and ciliates (*Tetrahymena thermophile)* local adaptation to marked temperature differences was facilitated by adaptive dispersal decisions of distinct thermal phenotypes to their matching thermal habitats (Bestion et al., 2015; Jacob et al., 2017) may thus be examples of the type of solution that contemporary disturbed populations will increasingly express or evolve. This hypothesis warrants further testing.

### 4.2 Strength of divergent selection, assortative mating, and speciation

Our simulations confirmed that strong spatial and temporal environmental heterogeneity increases the pressure to specialize (e.g., Kisdi, 2002). Genotypic mismatch indeed decreased when populations experienced stronger environmental changes, particularly when adaptation was achieved via divergent natural selection. Interestingly, under conditions favoring the evolution of locally adapted specialists, matching habitat choice had a higher capacity at eliminating maladaptive hybrids from the meta‐population than natural selection. Additionally, local adaptation and evolutionary stability of the meta‐populations collapsed in the scenarios of natural selection when reproduction was global, due to the strong genetic costs of producing maladaptive hybrids (the rarer genotypes produces proportionally more hybrids, so fitness is positive frequency dependent). These results corroborate recent modeling showing that the negative effects of random gene flow (e.g., preventing local adaptation via migration load), as due to random dispersal or global mating here, dominate its positive effects (e.g., spreading beneficial mutations) whenever conditions of patch selection favored the evolution of specialists (Bourne et al., 2014).

Adaptive population genetic differentiation will indirectly promote positive assortative mating (Jiang, Bolnick, & Kirkpatrick, 2013) when reproduction happens within the patch of settlement. Because matching habitat choice generally promotes genetic divergence through enhanced spatial isolation of the different genotypes, an important implication of our results is that matching habitat choice could speed up the genetic divergence among populations and the evolution of assortative mating, thereby increasing the feasibility of sympatric speciation (Fry, 2003; Gavrilets, 2014; Kirkpatrick & Ravigné, 2002). When the environmental changes were strong and mating was local, we observed maximal population genetic divergence at both functional and neutral genotypes (*Q*st and n*Q*st = 1.00, Figure 4k,l), and the absence of intermediate, hybrid genotypes (Figure 3o), despite the fact that almost all individuals switched between patches each generation (migration rate approximately 1.0). A very similar result is observed for mild environmental changes and local mating: a high functional *Q*st and a high neutral *Q*st. These high values indicate a high level of reproductive isolation between specialists on an environmental gradient. Hence, similarly to experimental results of Rice (1985) where differences in habitat preference played a more important role in generating reproductive isolation than assortative mating, our model could be interpreted as a model of sympatric speciation, driven almost exclusively by the nonrandom settlement of dispersers, and in the absence of mate choice. Previous models have also shown the importance of habitat choice for speciation (see Fry, 2003; Ravigné et al., 2009) when habitat choice and ecological performance were coded by different genetic loci (“two‐trait” models, cf. Rice & Hostert, 1993), such that recombination can limit the build‐up of reproductive isolation between ecologically diverged populations due to breakdown of genetic linkage disequilibrium (Felsenstein, 1981). As matching habitat choice is based on performance and therefore only on the underlying functional loci and not on some divergence in habitat preference alleles (a “one‐trait” model, cf. Rice & Hostert, 1993), such recombination is avoided and the evolution of reproductive isolation is facilitated. Matching habitat choice therefore shares a conceptual similarity with a “magic trait” (Edelaar et al., 2008; Gavrilets, 2014; Servedio, Van Doorn, Kopp, Frame, & Nosil, 2011), which is a functional trait that is under divergent natural selection and at the same time causes reproductive isolation. To the extent that matching habitat choice is actually involved in the process of local adaptation and mating is local, it should always contribute to a restriction of gene flow between locally adapting populations. In addition, matching habitat choice is also a so‐called one‐allele trait (Berner & Thibert‐Plante, 2015; Gavrilets, 2014), in the sense that it is selected for in the same direction in all individuals, independent of their ecological characteristics and (sub)population membership. This should further enhance the capacity to promote speciation. Nonetheless, solid empirical evidence supporting a role for matching habitat choice in speciation is still lacking, as far as we know.

### 4.3 Why is matching habitat choice not more prevalent in natural systems?

Our simulations reveal that matching habitat choice can come with large advantages: it allows for improved local performance, even in a context of strong environmental change or global mating. It shares these advantages with adaptive plasticity. One might therefore expect it to be equally common in nature, but this has yet to be confirmed (Edelaar et al., 2008).

One reason for this is that there might be limitations to its evolution in nature. First, the range of species able to evolve matching habitat choice might be restricted. This is because adaptive dispersal decisions usually imply that organisms should be sufficiently mobile to disperse over a relevant range of environmental variation and be equipped with the adequate sensory apparatus and cognition abilities to assess available settlement options in terms of relative ecological performance (Bernays & Wcislo, 1994). Second, using matching habitat choice may in fact be quite costly. Our models deliberately imposed no direct costs associated with plasticity and dispersal traits because we were interested in the population consequences of different adaptive mechanisms rather than in their (co)evolution per se. However, in reality both plasticity (Auld et al., 2010) and dispersal (Bonte et al., 2012) encompass a number of costs, and the relative importance of these costs will influence the evolution of the adaptive mechanisms: evolution typically favors the less costly solution (Edelaar et al., 2017). Third, kin competition and inbreeding are two major drivers of dispersal evolution (Gandon, 1999; Hamilton & May, 1977), but the spatial clustering of similar and therefore potentially related genotypes might actually be favored by matching habitat choice. Kin competition will act in our model, but its importance remains unknown. Therefore, the negative demographic and genetic consequences (e.g., inbreeding depression and negative growth leading to local extinctions) that matching habitat choice may have need to be investigated in more detail, especially as other mechanisms of adaptation (plasticity, natural selection) generally favor low dispersal rates (philopatry) and therefore may also suffer from kin competition and inbreeding. Examining the population consequences of the different modeled mechanisms with varying costs of kin competition and genetic load would be an interesting extension of our models (see, e.g., Henry, Coulon, & Travis, 2015).

Alternatively, the existence of matching habitat choice may be underestimated in natural systems because without manipulation of the phenotypes and observing the consequences for dispersal, observed local environment‐phenotype matching as those depicted in our figures may be mistakenly interpreted as typical outputs of plasticity or natural selection (Figure 1; Camacho, Canal, & Potti, 2015; Edelaar & Bolnick, 2012). Given that matching habitat choice may still operate when plasticity is constrained, and that it favors the maintenance of genetic variation and the evolution of reproductive isolation, it is very worthwhile to consider whether matching habitat choice might have contributed to patterns of observed local adaptation, and if so to test for matching habitat choice empirically.

### 4.4 Further model assumptions and potential extensions

Specific simulation results depend on specific model assumptions. As is true for any theoretical model, different choices could have been made for this study, and some of our assumptions may not have direct equivalents in nature. However, these choices were made to simplify the models, to isolate and highlight certain effects of interest, and to avoid effects that were not of interest. Overall, we believe that our assumptions will have mostly quantitative instead of qualitative effects on our most important inferences, some of which are in line with classical results obtained by other means. In addition to the arguments provided in the Methods section, we discuss the most important assumptions here. First, we did not implement any spatial structure to the patches and allowed individuals to be free to move anywhere, and this may come across as unrealistic. However, this avoids genetic divergence by isolation by distance (Wright, 1943), which is a balance between diverging genetic drift and converging random gene flow and which is not of interest here. Also, when patches have distances between them, the probability of exploring a certain patch becomes a function of distance, but the shape of this exploration function and the scale of any spatial autocorrelation among patches then become important conditioning and complicating (if not arbitrary) choices (Armsworth, 2009). Second, we only modeled two types of environmental changes (mild and predictable vs. strong and unpredictable) without exploring all the range of possible options. Other studies showed that different adaptive responses are likely to evolve under different time scales, predictabilities, and magnitudes of environmental variations (e.g., Berdahl, Torney, Schertzer, & Levin, 2015; Botero, Weissing, Wright, & Rubenstein, 2015; Gabriel, Luttbeg, Sih, & Tollrian, 2005). For example, reversible plasticity is predicted to be more likely to evolve in response to frequent and predictable environmental changes while more stable conditions should favor adaptive tracking by phenotypes through natural selection (Botero et al., 2015). We chose this approach because initial simulations showed that a standard deviation of 10 for the environmental change distribution enables the maintenance of meta‐populations even in the absence of plasticity or matching habitat choice (data not shown). We also have modeled strong environmental changes (random alternation between two extreme “*environment*” values) to mimic conditions that foster speciation and to make it more comparable to the commonly investigated two‐patch models (Gavrilets, 2014). Third, we modeled small local population sizes with a maximum of 10 individuals per patch, which could promote the importance of genetic drift and therefore reduce that of natural selection. However, the dispersal between demes (as here) reduces the negative effects of drift on local adaptation (Blanquart, Gandon, & Nuismer, 2012). In their previous study and preliminary simulations, Edelaar et al. (2017) investigated the robustness of their results by modeling population sizes with 10 or 100 individuals in each of the 100 patches with the goal of perhaps reducing genetic drift and increasing the response to selection. However, these simulation runs gave similar output. Given these previous results and the fact that our independent replicates also gave very consistent results, we conclude that random genetic drift can be ignored. Last, individuals of the same population were mostly monomorphic regarding their dispersal and plasticity traits. However, there is increasing empirical evidence that within populations, dispersing and nondispersing types differ in many phenotypic traits forming correlated so‐called dispersal syndromes (Cote et al., 2010; Stevens et al., 2014), including their degree of phenotypic plasticity (Mathot, Wright, Kempenaers, & Dingemanse, 2012). An interesting follow‐up of this study would be to model the evolution of such syndromes and to evaluate their influence on meta‐population characteristics and stability (Elliott & Cornell, 2012; Fogarty, Cote, & Sih, 2011).

## 5 CONCLUSION

This study confirms that the ecological and evolutionary consequences for meta‐populations can greatly differ depending on the mechanism acting in response to environmental change. Particularly, it emphasizes that adaptive directed gene flow as due to matching habitat choice can be as (or even more) powerful in leading to increase in local adaptation, genetic variation, population genetic structure, and reproductive isolation, compared to more “classical” mechanisms like natural selection and plasticity. It also shows that divergent natural selection and matching habitat choice can easily result in very similar if not identical empirical patterns, making it very hard to infer the operation of one or another process based on pattern alone. We suggest that studies of local adaptation, after rejecting plasticity, should not simply accept the default explanation of divergent natural selection as the cause, without considering habitat choice as well. Given the relative scarcity of studies testing the evolutionary potential of matching habitat choice, we hope that our findings will stimulate future theoretical and empirical studies and applications of matching habitat choice, including conservation tools such as assisted dispersal, improving habitat network connectivity (Travis & Dytham, 2012), and maintenance of habitat variation that provides the preferred environment for distinct genotypes.

## CONFLICT OF INTEREST

None declared.

## AUTHOR CONTRIBUTIONS

MN and PE designed the study and data analyses. MN performed all modeling and data analyses. MN and PE wrote the manuscript.
