# Supplementary material for: Comparing the consequences of natural selection, adaptive phenotypic plasticity, and matching habitat choice for phenotype–environment matching, population genetic structure, and reproductive isolation in meta‐populations
Source: Ecol Evol. 2018 Mar 13;8(8):3815–27. doi: 10.1002/ece3.3816 (PMC5916293; doi:10.1002/ece3.3816)
Supplement: Supplementary file 1 [file ECE3-8-3815-s001.doc]

**Supporting Information** of the manuscript entitled:

Comparing the consequences of natural selection, adaptive phenotypic plasticity and matching habitat choice for phenotype-environment matching, population genetic structure and reproductive isolation in meta-populations

**Supporting Information SI 1:** Table S1. Variables and parameterisation used for simulations

**Supporting Information SI 2:** Figure S2. Example visual output of simulations

**Supporting Information SI 3:** Table S3. Numerical results of the simulations

**Supporting Information SI 4:** Data S1. NetLogo code of the individual-based simulation model

**Table S1.** Variables and parameterisation used for simulations

| **variable** | **range value** | | | | | **description** |
| --- | --- | --- | --- | --- | --- | --- |
| **parameters** | identical for all scenarios | | | | |  |
| *standard-deviation-change* | 10 | | | | | Determines the degree of temporal predictable environmental change: *Environment* _t+1_ = *Environment* _t_ + N ~ (0, 10). |
| *mean-mutational-change* | 0.01 | | | | | Mutation is modelled by extracting a pseudo number from an exponential decay distribution with a mean of 0.01 and adding or subtracting it from the inherited trait with equal probability. |
| **dynamic variables** | identical for all scenarios | | | | |  |
| *environment* | [0, 360º] vs. 40º or 220º | | | | | Single variable describing the features of the habitat patch when the environment changes in a mild and predictable way ([0, 360º]) vs. strongly in an unpredictable way (40º or 220º). |
| *phenotype* | [0, 360º] | | | | | Individual trait. Without plasticity, *phenotype* = *genotype.* |
| *phenotypic mismatch* | [0, 360º] | | | | | Absolute value of (*environment* - *phenotype*). |
| *genotypic mismatch* | [0, 360º] | | | | | Absolute value of (*environment* - *genotype*). |
| **individual heritable traits** | scenario I | scenario II | scenario III | scenario IV | scenario V |  |
| *genotype* | identical for all scenarios [0, 360º] | | | | | Genotypic value for a functional trait. Affects the *phenotype* to utilize the *environment.* |
| *neutral genotype* | identical for all scenarios [0, 360º] | | | | | Genotypic value for a neutral trait. |
| *plasticity potential* | 0 | 0 | 1 | 1 | 0 | Proportion by which maximum phenotypic mismatch can be eliminated by plasticity. |
| *plasticity habitat sensitivity* | 0 | 0 | 1 | 1 | 0 | Proportion of lower potential values of the environment that is excluded from the assessment of the patch in which an individual develops. |
| *dispersal potential* | [0, 0.1] | [0.9, 1] | 0 | [0, 1] | 1 | Probability that a given non-natal patch is included in the set of patches that is prospected and to which an individual could potentially disperse. |
| *dispersal habitat sensitivity* | 0 | 0 | 0 | 0 | 1 | Proportion of lower potential values of the environment that is excluded from the assessment of each of the patches to which an individual could potentially disperse. |

**Figure S2: Examples of visual output of Netlogo simulations** at start (A) (comparable for all scenarios) and after 1000 generations for 3 out of 20 scenarios: matching habitat choice with directed dispersal (B), adaptive plasticity with no dispersal (C) and divergent selection with low random dispersal (D). In all examples, reproduction occurs within patches and the environmental changes between generations are mild and predictable. Our meta-population consists of 100 patches (white circles) assigned with a random *environment* at start (visualize by a long grey bar within patch). Individuals settling within these patches (and their *genotype* value) are represented with small bars (different genotypes are represented with a unique colour). If genotypes are locally adapted, values *environment* and *genotype* are lined-up (as in B). The frequency distributions of the *environment*, *phenotype* and *genotype* at start (A) or after 1000 generations (B-D) are shown below the meta-populations (range of *environments* on the x-axis from 0 to 360).


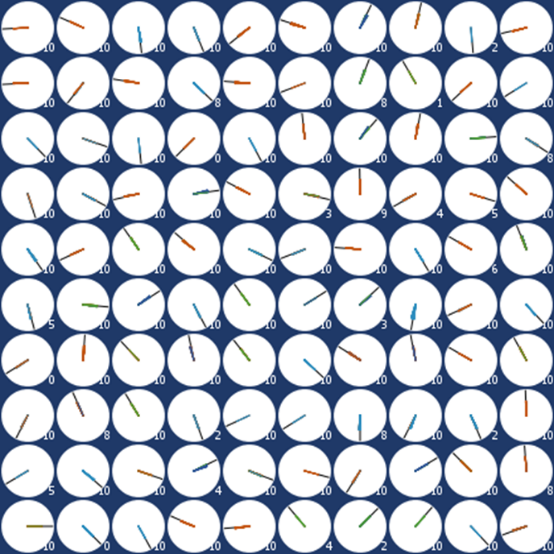

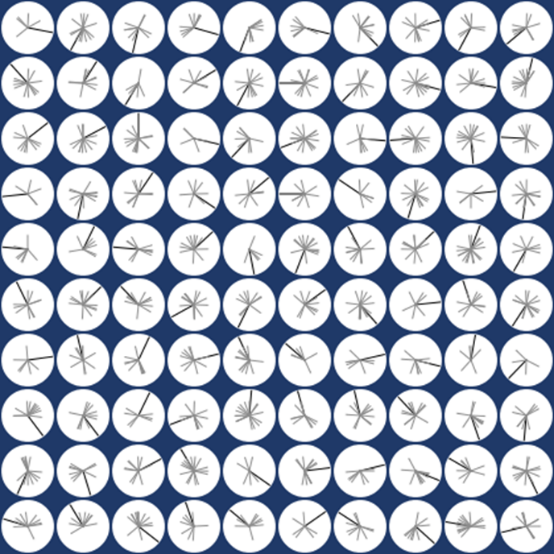

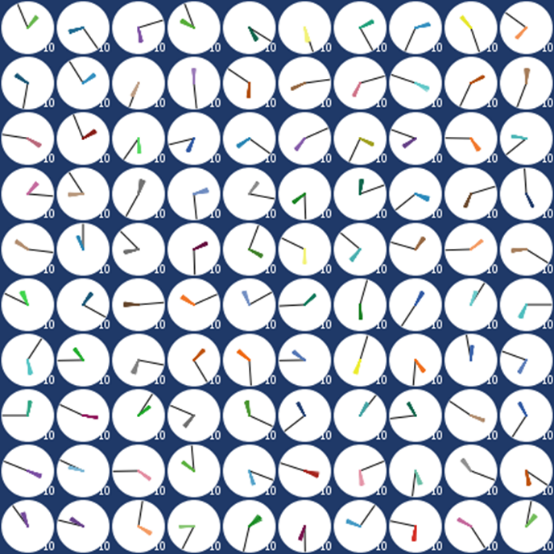

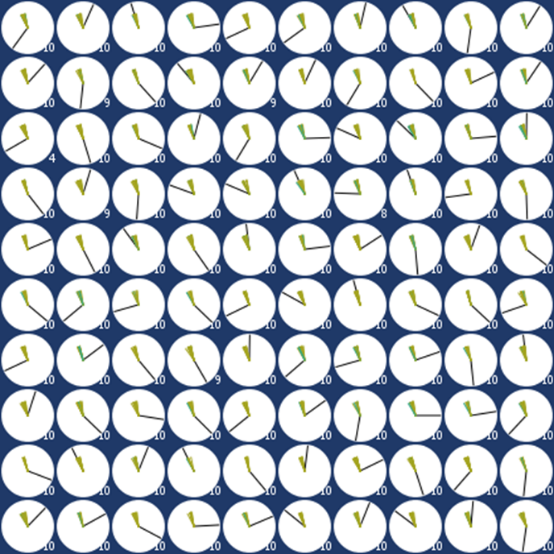

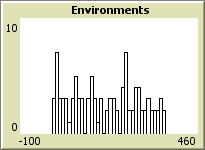

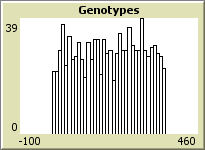

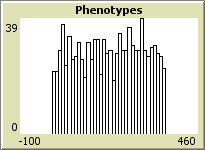

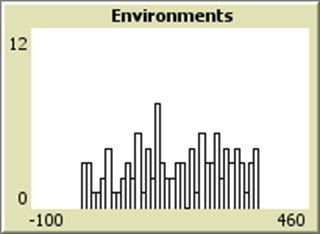

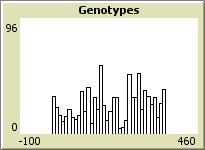

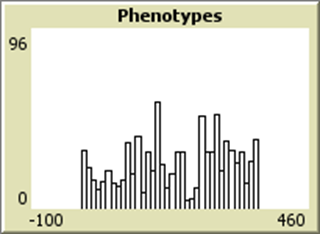

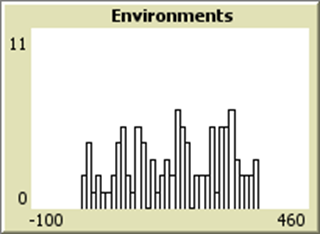

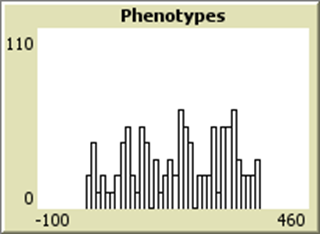

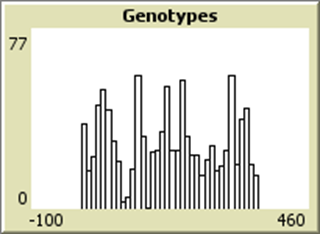

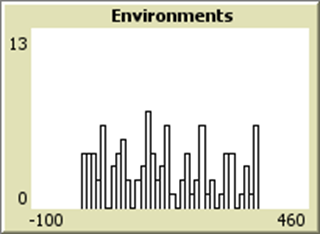

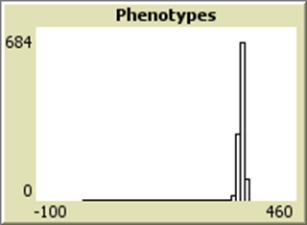

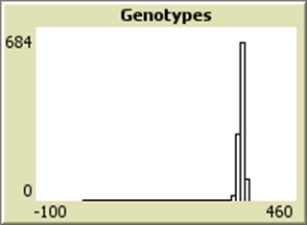


1. at start

B. matching habitat choice

with directed dispersal

C. adaptive plasticity with no dispersal

D. natural selection with low random dispersal

Trait values

Trait values

Trait values

Trait values

**Table S3. Numerical results of the simulations** for the 5 scenarios considered: natural selection with low dispersal (I), natural selection with high dispersal (II), adaptive plasticity without dispersal (III), adaptive plasticity with dispersal (IV) and matching habitat choice (V). We present the consequences of these distinct scenarios for the meta-population characteristics (average phenotypic (∆p) and genotypic (∆g) mismatch; genetic structure for a functional (Qst) or neutral (nQst) trait) when environmental changes are modelled as either mild and predictable or strong and unpredictable and when individuals reproduce either locally or globally. The distributions of simulation results are summarized by their posterior modes and their 95% credible intervals.

| **scenario** | | **reproduction** | **∆p (95% CrI)** | **∆g (95% CrI)** | **Qst (95% CrI)** | **nQst (95% CrI)** |
| --- | --- | --- | --- | --- | --- | --- |
| mild and predictable environmental changes | | | | | | |
| nat. sel., low disp. | I | local | 35 (34, 179) | 35 (34, 179) | 0.55 (0.32, 0.66) | 0.35 (0.33, 0.63) |
| nat. sel., high disp. | II | local | 89 (84, 166) | 89 (84, 166) | 0.45 (0.33, 0.46) | 0.33 (0.33, 0.34) |
| plast., no disp. | III | local | 0 (0, 3) | 116 (107, 131) | 0.85 (0.78, 0.94) | 0.97 (0.91, 1.00) |
| plast., disp. | IV | local | 0 (0, 0) | 175 (83, 178) | 0.33 (0.33, 0.34) | 0.33 (0.33, 0.34) |
| match. hab. choice | V | local | 1 (0, 2) | 1 (0, 2) | 1.00 (0.91, 1.00) | 0.88 (0.69, 0.99) |
| nat. sel., low disp. | I | global | 115 (81, 156) | 115 (81, 156) | 0.45 (0.41, 0.48) | 0.34 (0.33, 0.34) |
| nat. sel., high disp. | II | global | 85 (85, 164) | 85 (85, 164) | 0.43 (0.37, 0.47) | 0.33 (0.33, 0.34) |
| plast., no disp. | III | global | 0 (0, 1) | 100 (96, 187) | 0.35 (0.34, 0.49) | 0.34 (0.33, 0.53) |
| plast., disp. | IV | global | 0 (0, 0) | 94 (86, 177) | 0.33 (0.33, 0.34) | 0.33 (0.33, 0.34) |
| match. hab. choice | V | global | 0 (0 , 3) | 0 (0 , 3) | 1.00 (0.94, 1.00) | 0.34 (0.33, 0.37) |
| strong and unpredictable environmental changes | | | | | | |
| nat. sel., low disp. | I | local | 7 (5, 12) | 7 (5, 12) | 0.75 (0.74, 0.89) | 0.79 (0.65, 0.83) |
| nat. sel., high disp. | II | local | 6 (4, 10) | 6 (4, 10) | 0.81 (0.78, 0.93) | 0.88 (0.70, 0.91) |
| plast., no disp. | III | local | 0 (0, 6) | 116 (108, 133) | 0.89 (0.82, 0.97) | 0.94 (0.86, 1.00) |
| plast., disp. | IV | local | 0 (0, 0) | 90 (82, 183) | 0.33 (0.33, 0.34) | 0.34 (0.33, 0.34) |
| match. hab. choice | V | local | 1 (1, 1) | 1 (1, 1) | 1.00 (1.00, 1.00) | 1.00 (0.53, 1.00) |
| nat. sel., low disp. | I | global | 82 (66, 98) | 82 (66, 98) | 0.33 (0.33, 0.34) | 0.33 (0.33, 0.34) |
| nat. sel., high disp. | II | global | 91 (69, 116) | 91 (69, 116) | 0.33 (0.33, 0.34) | 0.34 (0.33, 0.34) |
| plast., no disp. | III | global | 0 (0, 3) | 97 (85, 199) | 0.34 (0.33, 0.48) | 0.34 (0.33, 0.51) |
| plast., disp. | IV | global | 0 (0, 0) | 90 (89, 219) | 0.33 (0.33, 0.34) | 0.33 (0.33, 0.34) |
| match. hab. choice | V | global | 1 (1, 1) | 1 (1, 1) | 0.34 (0.33, 0.34) | 0.34 (0.33, 0.34) |

**Supporting Information S4. NetLogo code of the individual-based simulation model.**

; This program was developed using NetLogo 5.0 (http://ccl.northwestern.edu/netlogo)

; It implements the model described in:

; Authors: Marion Nicolaus and Pim Edelaar

; Article: “Comparing the consequences of natural selection, adaptive phenotypic plasticity and matching habitat choice for phenotype-environment matching, population genetic structure and reproductive isolation in meta-populations”

; Ecology & Evolution (2017)

; The program is free of use for research and education. If you use this program or model

; for your own research, please refer to our paper as described above.

;------------------------------------------------------------------------------------------------------------------------------------------

Breed [ clock-circles clock-circle ] ; for display purposes only

Breed [ habitats habitat ]

Breed [ individuals individual ]

;variables:

globals [ seed simulation ]

habitats-own [ true-environment-here

estimated-environment

habitat-quality ;the "heading" of the habitat-individual is a built-in variable used as the 'environment' value of the habitat.

local-genotypes

local-phenotypes

local-neutral-genotypes

habitat-value ]

individuals-own [phenotype

neutral-heading

genotype1 ; genotype of the focal individual

genotype2 ; genotype of individual’s partner

x1

y1

x2

y2

mean-x

mean-y

neutral-x1

neutral-y1

neutral-x2

neutral-y2

neutral-mean-x

neutral-mean-y

partner

mean-heading ; mean of the individual and its partner

neutral-mean-heading ; mean of the individual and its partner

reproductive-potential

N-descendence

plasticity-habitat-sensitivity1 ; trait of the focal individual

dispersal-habitat-sensitivity1 ; trait of the focal individual

plasticity-potential1 ; trait of the focal individual

dispersal-potential1 ; trait of the focal individual

plasticity-habitat-sensitivity2 ; trait of the individual’s partner

dispersal-habitat-sensitivity2 ; trait of the individual’s partner

plasticity-potential2 ; trait of the individual’s partner

dispersal-potential2 ; trait of the individual’s partner

plasticity-habitat-sensitivity ; trait of the offspring (mean of the individual + partner)

dispersal-habitat-sensitivity ; trait of the offspring (mean of the individual + partner

plasticity-potential ; trait of the offspring (mean of the individual + partner

dispersal-potential ; trait of the offspring (mean of the individual + partner

my-habitat] ; the "heading" of the individual is a built-in variable used as the 'genotype' of the individual.

;----------------- INTERFACE BUTTONS -----------------------

to SETUP

clear-all

reset-ticks

random-seed seedN

ask patches

[ set pcolor 103 ;for display purposes only

sprout-habitats 1

[ set heading random-float 360 ;All habitats start with a random angle (i.e. a random environment value between 0 and 360). If changes are modelled as strong and unpredictable we set heading as “one-of [40 220]”

set size 0.9 ;Display purposes only

set color black ;Display purposes only

set shape "line half" ] ] ;Display purposes only

create-individuals 1000

[ move-to one-of habitats ;Individuals are initially distributed randomly across the available patches.

;Individuals start with random traits (and genotype = phenotype) ]

set heading random-float 360 ; genotype of the individual

set neutral-heading random-float 360 ; neutral genotype of the individual

set phenotype heading

set plasticity-potential 0 ; fix value depending on the scenario modelled

set plasticity-habitat-sensitivity 0 ; fix value depending on the scenario modelled

set dispersal-potential 1 ; fix value depending on the scenario modelled

set dispersal-habitat-sensitivity 1 ; fix value depending on the scenario modelled

set shape "line half" ;Display purposes only

set size 0.5 ;Display purposes only

set color gray ] ;Display purposes only

update-plots

tick-advance 1

ask patches ;Display purposes only.

[ sprout-clock-circles 1 [ set shape "circle" set size 0.95 set color white ] ] ;Display purposes only.

end

;************************************************************************************

to Thousand-Generations

repeat 999 ; number of generations the simulation will run after initialisation

[ One-Generation ]

end

;************************************************************************************

to One-Generation

Calculate-reproductive-potential ; see submodels below.

Negative-density-dependent-mortality ; see submodels below.

calculate-local-parameters ; see below.

update-plots

ask patches ; Display purposes only.

[ set plabel-color white set plabel count individuals-here ] ;Display purposes only.

ask individuals ;"adults"

[if count individuals-here > 1 ; reproduction here is LOCAL and is allowed only if there are at least 2 individuals in the patch

[Reproduction ] ;See submodels below. It simulates non-overlaping generations, i.e. individuals reproduce...

Die ] ;... and then die.

Environmental-change ;See submodels below.

ask individuals ;"offspring"

[ ifelse what-first? = "develop"

[ Development ;See submodels below.

Dispersal ] ;See submodels below.

[ Dispersal ;See submodels below.

Development ] ] ;See submodels below.

tick-advance 1

end

;************************************************************************************

;---------------------SUBMODELS------------------------------------------

to Development

let true-mismatch-before-development abs ( subtract-headings mean [ heading ] of habitats-here phenotype )

let my-plasticity-habitat-sensitivity plasticity-habitat-sensitivity

ask habitats-here

[ set true-environment-here heading

;==================================================================================

;Following code introduces noise on the estimated environment value by turning the heading of the

;habitat first left and then right (lower the habitat-sensitivity larger the left and right turns)

;e.g. if plasticity-habitat-sensitivity = 0 the environment value turns left (lt) 180º and then right (rt) by ; a random-float number between 0 and 360º.

;The "/2" is needed to first turn left yielding half the "noise", then turn right to give all the "noise".

lt ( 360 * ( ( 1 - my-plasticity-habitat-sensitivity ) / 2 ) )

rt random-float ( 360 * ( 1 - my-plasticity-habitat-sensitivity ) )

set estimated-environment heading

;==================================================================================

set heading true-environment-here ] ;restores the heading to the true environment value of the habitat patch.

let mismatch subtract-headings mean [ estimated-environment ] of habitats-here heading ;'mismatch' reports the (estimated) need of change of the genotype (and its sign) to match the environment

let genotype heading ;to preserve the current heading (this is a trick to be able to use rt (right turn) below).

ifelse abs mismatch > ( plasticity-potential * 180 ) ;i.e. if plasticity-potential is smaller than needed (i.e. smaller than mismatch)...

;...then use all the available plasticity-potential in the right direction:

[ ifelse mismatch < 0

[ lt ( plasticity-potential * 180 ) ]

[ rt ( plasticity-potential * 180 ) ] ]

;...otherwise, jump to have a phenotype = estimated-environment.

[ set heading mean [ estimated-environment ] of habitats-here ]

set phenotype heading

set heading genotype ;This restores the heading (the genotype) of the individual.

end

;***********************************************************************************

to Dispersal

let true-mismatch-before-dispersal abs ( subtract-headings mean [ heading ] of habitats-here phenotype )

let my-dispersal-potential dispersal-potential

let my-phenotype phenotype

let my-dispersal-habitat-sensitivity dispersal-habitat-sensitivity

let my-natal-patch patch-here

ask habitats-here ;i.e. natal patch

[ set true-environment-here heading

lt ( 360 * ( ( 1 - my-dispersal-habitat-sensitivity ) / 2 ) )

rt random-float ( 360 * ( 1 - my-dispersal-habitat-sensitivity ) )

set estimated-environment heading

set heading true-environment-here ;restore the heading to the true environment value of the habitat patch

set habitat-quality -1 * abs ( subtract-headings estimated-environment my-phenotype )

ask other habitats ;i.e. non-natal patches (this is necessary because in NetLogo an individual cannot ask something to all patches at once.

[ set habitat-quality -999

if random-float 1 < my-dispersal-potential ;the larger the dispersal-potential, the larger the set of patches evaluated for quality.

[ set true-environment-here heading

lt ( 360 * ( ( 1 - my-dispersal-habitat-sensitivity ) / 2 ) )

rt random-float ( 360 * ( 1 - my-dispersal-habitat-sensitivity ) )

set estimated-environment heading

set heading true-environment-here ;Restores the heading to the true environment value of the habitat patch.

set habitat-quality -1 * abs ( subtract-headings estimated-environment my-phenotype ) ] ] ] ; the higher the mismatch, the lower (more negative) the habitat quality

move-to max-one-of habitats [ habitat-quality ] ;The individual disperses to the habitat patch (perceived to be) with an environment closest to its phenotype.

if patch-here != my-natal-patch ;Display purposes only.

end

;************************************************************************************

to Calculate-reproductive-potential ; Note that here all costs are set to 0

ask individuals

[ set reproductive-potential 1 -

( plasticity-potential * plasticity-potential-costs ) -

( plasticity-habitat-sensitivity * plasticity-habitat-sensitivity-costs ) -

( dispersal-potential * dispersal-potential-costs ) -

( dispersal-habitat-sensitivity * dispersal-habitat-sensitivity-costs ) -

( abs ( subtract-headings mean [ heading ] of habitats-here phenotype ) / 180 ) ;This reports the mismatch between the phenotype and the 'environment' (the heading of the habitat).

;Since the maximum distance between two positions in a circle is 180º, "/180" makes the 'mismatch' between 0 and 1.

;Since there is a single "habitat" in each patch, "mean [ heading ] of habitats-here" reports the environment value of the patch. ]

end

;************************************************************************************

to Negative-density-dependent-mortality

ask habitats

[ if any? individuals-here

[ let Nsurplus count individuals-here - 10 ;'Nsurplus' reports how many individuals should die.

if Nsurplus > 0

[ repeat Nsurplus

[ ask min-one-of individuals-here [ reproductive-potential ] ;the individual with lowest 'reproductive-potential' dies sequentially as often as indicated by 'Nsurplus'.

[ die ] ] ] ] ]

end

;************************************************************************************

to Reproduction

set genotype1 heading ; genotype of the focal individual

set x1 sin heading

set y1 cos heading

set neutral-x1 sin neutral-heading

set neutral-y1 cos neutral-heading

set plasticity-potential1 plasticity-potential ; trait of the focal individual

set plasticity-habitat-sensitivity1 plasticity-habitat-sensitivity ; trait of the focal individual

set dispersal-potential1 dispersal-potential ; trait of the focal individual

set dispersal-habitat-sensitivity1 dispersal-habitat-sensitivity ; trait of the focal individual

set partner one-of other individuals-here

set genotype2 [heading] of partner ; genotype of the partner

set x2 sin [heading] of partner

set y2 cos [heading] of partner

set neutral-x2 sin [neutral-heading ] of partner

set neutral-y2 cos [neutral-heading ] of partner

set plasticity-potential2 [plasticity-potential] of partner ; trait of the partner

set plasticity-habitat-sensitivity2 [plasticity-habitat-sensitivity] of partner ; trait of the partner

set dispersal-potential2 [dispersal-potential] of partner ; trait of the partner

set dispersal-habitat-sensitivity2 [dispersal-habitat-sensitivity] of partner ; trait of the partner

set mean-x ( x1 + x2 ) / 2

set mean-y ( y1 + y2 ) / 2

set mean-heading atan mean-x mean-y

set neutral-mean-x ( neutral-x1 + neutral-x2 ) / 2

set neutral-mean-y ( neutral-y1 + neutral-y2 ) / 2

set neutral-mean-heading atan neutral-mean-x neutral-mean-y

set N-descendence 0 ;'Ndescendence' is the number of offspring the individual will have. It could be 0, 2 or 3.

if random-float 1 < reproductive-potential [ set N-descendence N-descendence + 2 ]

if random-float 1 < reproductive-potential [ set N-descendence N-descendence + 1 ]

hatch N-descendence

;the functional and neutral genotypes of the individual are inherited from the two parents and mutate

set heading mean-heading + ( one-of [-1 1] ) * random-exponential ( 0.01 * 180 )

set neutral-heading neutral-mean-heading + ( one-of [-1 1] ) * random-exponential ( 0.01 * 180 )

set phenotype heading

;The rest of the genetic traits of the individual are inherited from the parents without mutation

set plasticity-potential (plasticity-potential1 + plasticity-potential2) / 2

set plasticity-habitat-sensitivity (plasticity-habitat-sensitivity1 + plasticity-habitat-sensitivity2) / 2

set dispersal-potential (dispersal-potential1 + dispersal-potential2) / 2

set dispersal-habitat-sensitivity (dispersal-habitat-sensitivity1 + dispersal-habitat-sensitivity2) / 2

;then, these genetic traits are forced to remain between 0 and 1:

if plasticity-potential < 0 [ set plasticity-potential 0 ]

if plasticity-potential > 1 [ set plasticity-potential 1 ]

if plasticity-habitat-sensitivity < 0 [ set plasticity-habitat-sensitivity 0 ]

if plasticity-habitat-sensitivity > 1 [ set plasticity-habitat-sensitivity 1 ]

if dispersal-potential < 0 [ set dispersal-potential 0 ]

if dispersal-potential > 1 [ set dispersal-potential 1 ]

if dispersal-habitat-sensitivity < 0 [ set dispersal-habitat-sensitivity 0 ]

if dispersal-habitat-sensitivity > 1 [ set dispersal-habitat-sensitivity 1 ]

]

end

;************************************************************************************

to Environmental-change

ask habitats

[ rt random-normal 0 10 ]

; here the environmental changes are within 10 SD of the habitat value. If changes are modelled as alternative between 2 extreme values (e.g. 40 or 220), changes are defined as one-of [40 220]

end

;************************************************************************************

to calculate-local-parameters ; used to calculate numerical outputs of simulations

ask habitats

[ if any? individuals-here

[ set local-genotypes ( list [heading] of individuals-here )

set local-phenotypes ( list [phenotype] of individuals-here )

set local-neutral-genotypes (list [neutral-heading] of individuals-here )

set habitat-value [heading] of habitats-here ]]

end
